# Origin of ferromagnetism in Cu-doped ZnO

**DOI:** 10.1038/s41598-019-39660-x

**Published:** 2019-02-21

**Authors:** Nasir Ali, Budhi Singh, Zaheer Ahmed Khan, Vijaya A. R., Kartick Tarafder, Subhasis Ghosh

**Affiliations:** 1 School of Physical Sciences, Jawaharlal Nehru University, New Delhi, 110067 India; 2 Inter-University Accelerator Centre, Aruna Asaf Ali Marg, New Delhi, 110067 India; 3 Semi-Conductor Laboratory, Department of Space, S.A.S. Nagar, Punjab India; 4 Department of Physics, National Institute of Technology Karnataka, Surathkal, 575025 India

## Abstract

It is widely reported during last decade on the observation of room temperature ferromagnetism (RTFM) in doped ZnO and other transition metal oxides. However, the origin of RTFM is not understood and highly debated. While investigating the origin of RTFM, magnetic ion doped oxides should be excluded because it is not yet settled whether RTFM is intrinsic or due to the magnetic ion cluster in ZnO. Hence, it is desirable to investigate the origin of RTFM in non-magnetic ion doped ZnO and Cu-doped ZnO will be most suitable for this purpose. The important features of ferromagnetism observed in doped ZnO are (i) observation of RTFM at a doping concentration much below than the percolation threshold of wurtzite ZnO, (ii) temperature independence of magnetization and (iii) almost anhysteretic magnetization curve. We show that all these features of ferromagnetism in ZnO are due to overlapping of bound magnetic polarons (BMPs) which are created by exchange interaction between the spin of Cu^2+^ ion and spin of the localized hole due to zinc vacancy $({V}_{Zn})$. Both the experimental and theoretical investigation show that the exchange interaction between Cu^2+^-Cu^2+^ ions mediated by ${V}_{Zn}$ is responsible for RTFM in Cu-doped ZnO.

## Introduction

Dilute magnetic oxides (DMOs) and dilute magnetic semiconductors (DMSs) are arguably the most interesting and intriguing magnetic materials and room temperature ferromagnetism (RTFM) in these materials is the most puzzling problem in contemporary solid state physics^1–5^. There have been innumerous reports on RTFM in ZnO films with Curie temperature (*T*_*C*_) above room temperature when ZnO doped with all possible dopants, even some reports claiming RTFM in undoped ZnO^6–12^. The original idea of obtaining DMOs was to dope with only magnetic ions such as Mn, Fe, Co, Ni similar in line with studies on magnetic ions doped III–V and II–VI semiconductors^13–16^. However, this idea was seriously challenged by raising a doubt in different experimental observations whether the RTFM is at all intrinsic^17–20^. As the experimental situation is crowded with somewhat contradictory and inconsistent results, we list here the main unresolved issues in DMOs, (1) DMOs do not order magnetically similar to that in ferromagnetic materials. It is now widely accepted that the ferromagnetic order depends on the presence of defect and this leads to results based on local probes like x-ray magnetic circular dichroism and x-ray absorption near edge spectroscopy doubtful^6,21–24^. (2) RTFM can be observed in extremely dilute systems. The magnetic cations concentration responsible for ferromagnetism is much below than the cation nearest neighbor percolation threshold (*p*_*c*_~0.198) for wurtzite ZnO^25^. Hence, all the exchange mechanisms such as super-exchange and double-exchange proposed to explain ferromagnetism are doubted as the separation between two dopant impurities is very large compared to the lattice constant of ZnO^26,27^. (3) Observation of RTFM in highly resistive sample lead to irrelevance of Rudermann-Kittel-Kausya-Yosida (RKKY) mechanism for ferromagnetism which has been proposed to explain RTFM^28,29^. (4) The ubiquitous feature like temperature independent magnetization has been observed in many reports, but no explanation has been provided till date and this raises a doubt whether the proposed exchange mechanism is relevant^30,31^. (5) The magnetization curves is almost anhysteretic which again shown in all reports however not explained^21,32^. One reason behind the ambiguity in the understanding of ferromagnetism in oxide may be due to overlooking these important features of RTFM. We have considered Cu-doped ZnO as the system for solving this problem. We have avoided using magnetic ions (Fe, Co, Ni, Mn) doped ZnO to rule out any possibility of magnetic ion cluster induced ferromagnetism. Moreover, Cu or Cu cluster or any oxide of Cu is not ferromagnetic. The RTFM in Cu-doped ZnO has been reported before^33–36^. Beyond a doubt it has been established that unpaired spin on Cu-site i.e. Cu^2+^ (3d^9^) is responsible for ferromagnetism, however it is not known what is the long-range interaction between Cu^2+^ is responsible for ferromagnetism with *T*_*C*_ above room temperature. Here we present a detailed experimental and theoretical investigation to identify the long-range interaction responsible for RTFM in ZnO.

## Results

**Figure 1:**
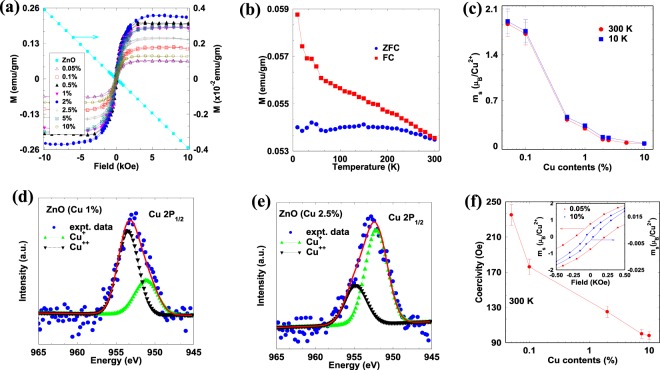
(**a**) Magnetization curves of ZnO doped with various concentration of Cu from 0.05% to 10%. It is clear that undoped ZnO is diamagnetic. (**b**) Zero fields cooled and field cooled magnetization of 2% Cu-doped ZnO film indicate that Curie temperature of Cu-doped ZnO is well above the room temperature. (**c**) Variation of saturated magnetic moment per Cu^2+^ ion at different doping concentration measured at 300 K and 10 K. Connecting lines are guide for eyes. (**d–e**) X-ray photoelectron spectroscopy of 1% and 2.5% Cu-doped ZnO films, respectively. (**f**) Dependence of coercivity on Cu-content in ZnO. Connecting line is guide for eyes. Inset shows the magnified hysteresis loop of 0.05% and 10% Cu-doped ZnO from −0.5 kOe to 0.5 kOe.

Figure 1a shows the magnetization curves of the undoped and Cu-doped ZnO films with varying doping concentrations from 0.05% to 10%, measured at 300 K using a Quantum Design SQUID magnetometer. The magnetization data at 10 K is shown in Supplementary Fig. S1. The bifurcation in zero field cooled (ZFC) and field cooled (FC) magnetization *vs*. temperature for 2.5% Cu-doped ZnO film as shown in Fig. 1(b) indicates that *T*_*C*_ is well above the room temperature. This is very important to note that all undoped ZnO which were grown under optimized conditions are diamagnetic as shown in Fig. 1a^35,37–40^. Hence, ferromagnetism in Cu-doped ZnO must be intrinsic and not due to only defect related^11,12^. Figure 1c shows the saturated magnetic moment $({m}_{s})$ per Cu^2+^ ion for different Cu concentration calculated from magnetization curves (Fig. 1a) indicating temperature independent ferromagnetic order at 300 K and 10 K. A ${m}_{s}$ of ~1.87 µ_B_/Cu^2+^ and ~1.72 µ_B_/Cu^2+^ are observed in 0.05% and 0.1% Cu-doped ZnO which matches well with the spin only moment per Cu^2+^ ion in 3*d*^9^ configuration *i*.*e*., ${\mu }_{eff}=g{\mu }_{B}\sqrt{S(S+1)}$ ~1.73 µ_B_/Cu^2+^^41,42^. The presence of such large value of ${m}_{s}$ at 0.05% doping concentration, which is very small compared to ${p}_{c}$ (~19.8%) required for long-range ferromagnetic order strongly infers the important role of intrinsic defects for long-range magnetic interaction among Cu atoms. The role of intrinsic defects on RTFM has already been emphasized in several reports^6,21,23^. The photoluminescence and absorption spectroscopy data as shown in Supplementary Fig. S3 revealed the presence of intrinsic defects in the Cu-doped ZnO through luminescence and absorption peaks at various energy levels other than the band-to-band transition. It is discussed in supplementary that below bandgap transition at ~3 eV can be attributed to zinc vacancy $({V}_{Zn})$ and broad peak at ~2.4 eV popularly known as green luminescence is due to defect complexes. It has been shown that oxygen vacancy $({V}_{O})$ and ${V}_{Zn}$ are the most dominant defects in ZnO when grown under either Zn- or O-rich conditions^43,44^. At higher doping concentration, the increase in the number of Cu atoms at the hexagonal wurtzite lattice can occupy adjacent cation sites which give antiferromagnetically coupled spins and hence reduces ${m}_{s}$ to a large extent at higher doping level (will be discussed in next section). The decrease in ${m}_{s}$ per Cu^2+^ ion with doping is further illustrated by X-ray photoelectron spectroscopy (XPS) measurements of 1% and 2.5% Cu-doped ZnO film as shown in Fig. 1d,e. XPS data revealed the presence of mixed oxidation state of Cu^2+^ and Cu^+^ ions corresponding to binding energy of 951.07 eV (952.12 eV) and 953.45 eV (954.65 eV) respectively in 1% (2.5%) Cu-doped ZnO films^33^. The + 1 oxidation state represents the presence of interstitial Cu with zero magnetic moment in the ZnO lattice. From the area corresponding to each peak (Supplementary Table I), one can conclude that the number of Cu^2+^ (Cu^+^) ions decreases (increases) with Cu doping.

Figure 1f shows that the coercivity of Cu-doped ZnO films at room temperature decrease with increasing Cu content. The hysteresis in magnetization curves of 0.05% and 10% Cu-doped ZnO films are shown in the inset of Fig. 1f. The magnetocrystalline anisotropy in Cu-doped ZnO film which arises mostly from spin-orbit (LS) coupling has been diminished by crystal filed splitting^45–47^, resulting an almost anhysteretic magnetization curves at higher doping concentration. In addition to this, absence of ferromagnetic domains in DMO’s is also responsible for anhysteretic magnetization. With Cu doping, the crystal field induced by Cu^2+^-Cu^2+^ interaction become much larger than the spin-orbit (LS) coupling. Due to this, coupling of *L* and *S* vectors are largely broken, so that the states no longer specified by *J* values. Further, the $2L+1$ sublevel belonging to a particular *L* which are degenerate in the free Cu^2+^ ion may now split by the crystal field and this diminishes the contribution of the orbital motion to the total magnetic moment of the Cu^2+^ ion.

**Figure 2:**
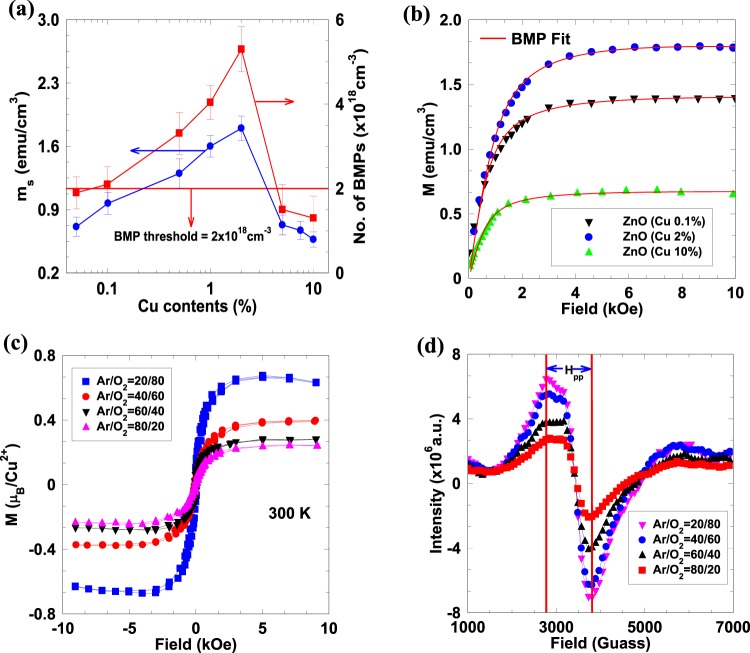
(**a**) Variation of saturated magnetic moment per cm^3^ and bound magnetic polarons concentration at various Cu doping from 0.05% to 10%. Connecting lines are guide for eyes. Horizontal line indicates the percolation threshold for bound magnetic polarons in the hexagonal wurtzite ZnO. (**b**) M–H curve fitted with BMP model. Symbols are for experimental data and the solid red line is a fit with BMP model. (**c**) Magnetization curves and (**d**) EPR spectra of 2% Cu-doped ZnO films grown at different Ar/O_2_ ratio.

Figure 2a shows ${m}_{s}$ per cm^3^ as a function of the Cu content in ZnO. As the Cu content increases from 0.05% to 10%, the ${m}_{s}$ per cm^3^ first increases and then decreases, reaching the maximum value of 2.48 emu/cm^3^ at 2% doping level. The role of intrinsic defects such as ${V}_{Zn}$ and ${V}_{O}$ to above RTFM in Cu-doped ZnO films have been investigated by depositing 2% Cu-doped ZnO films at different Ar/O_2_ ratio. The Ar-deficient atmosphere creates more ${V}_{Zn}$ compared to the Ar-rich atmosphere^33,43,44,48^. Figure 2c shows the magnetization data of 2% Cu-doped ZnO films grown at different Ar/O_2_ ratio. Increases in ${m}_{s}$ per Cu^2+^ ion with decreasing Ar/O_2_ ratio indicates that ${V}_{Zn}$ enhance the ferromagnetic order in Cu-doped ZnO. The presences of unpaired spins due to intrinsic defects within the Cu-doped ZnO films have been characterized by electron paramagnetic resonance (EPR) measurement. Figure 2d shows the EPR spectra of 2% Cu-doped ZnO films grown at different Ar/O_2_ ratio. All samples show the resonance peak due to ${V}_{Zn}^{-}$ at g = 2.01103 indicating the presence of ${V}_{Zn}^{-}$ under both Ar-rich and deficient conditions^49–51^. The increase in the intensity of resonance amplitude in Ar-deficient conditions indicates the presence of more spins due to ${V}_{Zn}^{-}$ available for magnetic interaction between Cu atoms. The concentration of spins can be given by the relation, $N\propto A{(\Delta {H}_{PP})}^{2}$^52^, where *N* is the spin concentration, *A* is the amplitude, and $\Delta {H}_{PP}$ is the peak-to-peak width of the EPR spectrum. The obtained value of *N* due to ${V}_{Zn}^{-}$ is found to be ~4 × 10^18^ cm^−3^. Finally, the temperature dependence resistivities of the Cu-doped ZnO films were studied and shown in Supplementary Fig. S4. Mott variable range hopping conduction in Cu-doped ZnO films indicates that the carriers are highly localized at intrinsic defect sites in Cu-doped ZnO^40,53,54^. Hence it can be concluded here that RKKY is not the origin of ferromagnetism in Cu-doped ZnO.

**Figure 3:**
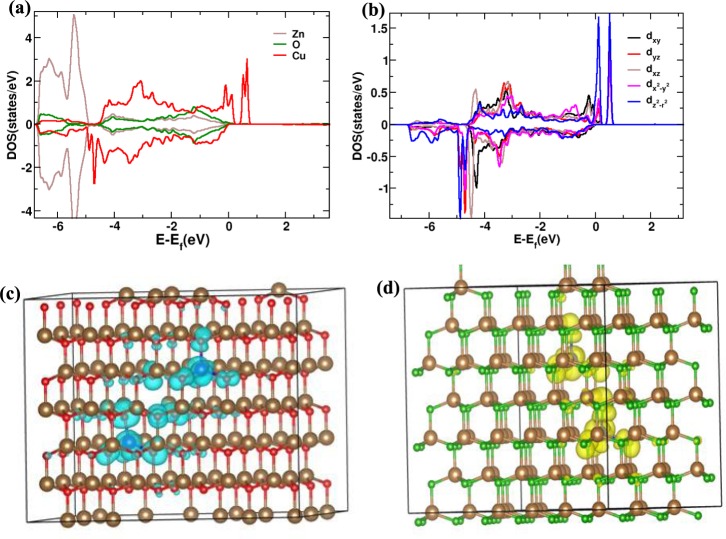
(**a**) Atom projected density of states in Cu-doped ZnO, (**b**) Cu-3d orbital density of states, (**c**) Significant amount of spin density distributed on neighboring O and (**d**) hole density due to V_Zn_.

For the better insight of magnetic coupling mechanism responsible for RTFM, we have performed first-principle DFT simulations using the full-potential Vienna *ab*-initio simulation package (VASP)^55,56^. The super cell used for DFT calculation is described in the methods section. The ferromagnetic stabilities were examined by comparing total energy difference $(\Delta E)$ between the ferromagnetic and antiferromagnetic configuration in different Cu-Cu separation. We found $(\Delta E=0)$, when the separations between two doped ions are 8 Å or more and the system behaves as a collection of isolated magnetic center instead of long-range ferromagnetically ordered spins. However, a weak antiferromagnetic coupling is found in case of relatively small separation distance less than 8 Å. The magnetization density overlap in two different situations is shown in Fig. 3 (a). Hence it appears that magnetic order is not possible in Cu-doped ZnO without intrinsic defect and situation is drastically changed as we introduce ${V}_{Zn}$ in our calculation. For 1% ${V}_{Zn}$, our DFT results show a strong ferromagnetic coupling energy of 40~60 meV whereas the presence of ${V}_{0}$ destroys the ferromagnetic order in Cu-doped ZnO. Our calculations indicate the partially filled Cu-e_g_ orbitals as shown in Fig. 3(b) giving rise to 0.64 ${\mu }_{B}$ magnetic moment on each Cu atom. Figure 3(c) shows a distribution of significant amount of spin density on neighboring O. The total magnetic moment of the supercell is 4 ${\mu }_{B}$. The spin density of the additionally created hole due to the ${V}_{Zn}$ is locally distributed between the doped Cu atoms (Fig. 3d), supporting interaction between spins on Cu atoms in relatively large distance of 7.5 Å^57^. Without Cu, ${V}_{O}$ does not play any role in magnetism. The total magnetic moment of the unit cell remains zero in presence of ${V}_{O}$ in ZnO. However, in presence of ${V}_{Zn}$, we found a small spin polarization with total magnetic moment in the super cell as 0.7 µ_B_ due to the unsaturated electrons in O atoms.

## Discussion

**Figure 4:**
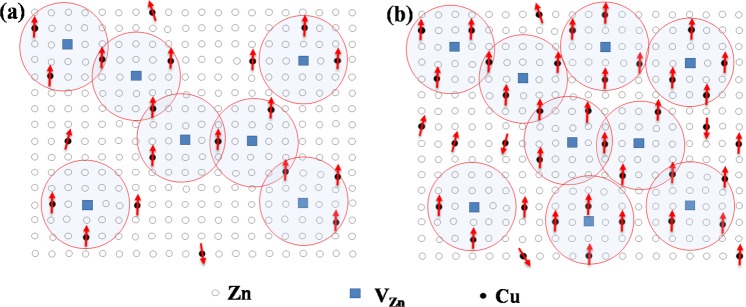
Schematic representation of long-range ferromagnetic order in Cu-doped ZnO mediated through bound magnetic polarons at (**a**) low doping concentration of 0.05% and (**b**) high doping concentration of 2% Cu-doped ZnO.

Based on the above findings, a possible explanation for the intrinsic magnetism can be found invoking an interaction between bound magnetic polarons (BMPs)^6,58,59^. The Bohr radius associated with ${V}_{Zn}$ is, $r=\varepsilon (\frac{{m}_{e}}{{m}^{\ast }}){a}_{0}$, $\varepsilon$ is the high-frequency dielectric constant, ${a}_{0}$ is Bohr’s radius, ${m}_{e}$ is free electron mass, ${m}^{\ast }$ is the effective mass of the carrier in ZnO *i*.*e*. ${m}^{\ast }=0.45{m}_{e}$^60,61^. We have measured $\varepsilon$ of ZnO film as a function of frequency at room temperature (Supplementary Fig. S4). Using $\varepsilon =21$, the confinement radius of the acceptor hole is found to be ~2.4 nm. The acceptor hole on ${V}_{Zn}$ creates a BMP by exchange interaction with Cu^2+^ ions. The average separation between two Cu^2+^ ions in 0.05% Cu-doped ZnO is ~2.1 nm (Supplementary Fig. S5), this means that at least two Cu^2+^ ions are available for magnetic exchange interaction in the vicinity of a ${V}_{Zn}$ acceptor hole. The exchange interaction between Cu^2+^ *via* localized hole on ${V}_{Zn}$ within a BMP takes the simple form as, $H=K\sum _{i}{s}_{i}.({S}_{i}+{S}_{i+1})$^58,59^, where *s* and *S* is the spin of hole and Cu^2+^ ion, respectively. *K* is the magnetic exchange interaction between the spin of Cu^2+^ *via* localized hole is 50 meV as obtained from our DFT calculations. The single BMP can be considered as an isolated ferromagnetic identity. As the concentration of the localized hole or ${V}_{Zn}$ increases; neighboring BMPs overlap and interact with each other *via* Cu^2+^ at Zn site. Percolation occurs when BMPs fill roughly ~14% of the space or samples size (*i*.*e*. ${p}_{c}$× packing fraction) as the ${p}_{c}$ and packing fraction in ZnO is 0.198 and 0.74, respectively^25^. The percolation threshold of BMP_S_ obtained for the appearance of long-range ferromagnetic order is (*i*.*e*. density of defects/cation density) ~5.6 × 10^−5^. Using the cation density of 3.94 × 10^22^ cm^−3^ in ZnO^6^, the defect concentration for percolation threshold is obtained as ~2 × 10^18^ cm^−3^. This concentration of ${V}_{Zn}$ as obtained from EPR measurement is more than the percolation threshold facilitating a continuum path with BMPs in the sample. The horizontal line in Fig. 2a indicates the ${V}_{Zn}$ concentrations for percolation threshold. The BMP concentrations can be obtained by fitting the magnetization curve using Eq.^62^.

1 $$M=n\,{m}_{s} {\mathcal L} (x)+{{\mathscr{X}}}_{m}H$$

where ${\mathcal L} (x)$ is the Langevin function with$\,x=\frac{{m}_{eff}H}{{k}_{B}T}$, ${k}_{B}$ is the Boltzmann constant, ${m}_{eff}$ is an effective spontaneous moment per BMP, *n* is the number of BMPs and ${{\mathscr{X}}}_{m}$ is the susceptibility of the matrix. The first term in Eq. 1 represents the contribution of the BMP’s and the term ${{\mathscr{X}}}_{m}H$ is the matrix contribution^62^. Figure 2b shows the fitting of M-H curves of the Cu-doped ZnO films. The obtained values of BMPs at different doping concentrations are greater than the BMP_S_ percolation threshold (as schematically shown in Fig. 4) which is required for long-range ferromagnetic order in wurtzite ZnO at room temperature (Fig. 2a). The overlapping of BMPs causes the alignment of Cu^2+^ spins (Fig. 4a), resulting ferromagnetic long chain of BMPs along the percolation path from one end of the sample to another end of sample resulting long-range ferromagnetic ordering between Cu^2+^ ions at room temperature. The corroboration of long-range ferromagnetic due to overlap of BMPs can be further established from the fact that the BMPs concentration follows the same trend as that of ${m}_{s}$ per cm^3^ for different Cu contents (Fig. 2a). Although, at low doping concentration (~0.05%) distance between two Cu^2+^ ions is very large but the overlap of BMPs leads to long-range ordering required for RTFM. As doping increases, more and more Cu^2+^ ions are accommodated inside BMPs with small separation resulting strong ferromagnetic order which becomes maximum at 2% doping level. After 2% doping level Cu impurities start occupying more cation sites of the wurtzite ZnO which give antiferromagnetically-coupled spins and hence reduces ${m}_{s}$ per cm^3^ to a large extent at higher doping. Similarly, at higher doping Cu^2+^ also starts occupying ${V}_{Zn}$ ions resulting decrease in BMP concentration with same trend as followed by ${m}_{s}$ per cm^3^. Finally, the temperature independent of ${m}_{s}$ in DMO’s can be explained by the fact that the parameters describing BMP-BMP interaction are temperature independent in contrary to polaron percolation in DMS as described by Kaminski *et al*.^63^. Hence the temperature independent magnetization, as observed in our experiment and many other reports^21,30^ can be explained by invoking the model put forward by Durst *et al*.^58^, according to which the overlap region of BMP does not depend on temperature. If the overlap region depends on temperature percolation path will be broken resulting disrupting in long-range ordering responsible for ferromagnetism in Cu doped ZnO.

In conclusions, the mechanism behind RTFM at a doping concentration much below than ${p}_{c}$ in Cu-doped ZnO has been discussed. A long-range ferromagnetic ordering has been developed, provided that there is a sufficient number of ${V}_{Zn}$ higher than the number of BMP_S_ required to achieve percolation threshold. First principle calculations show that a weak antiferromagnetic coupling is found in case of relatively small separation between Cu^2+^-Cu^2+^ pair. However, the exchange interaction between Cu^2+^-Cu^2+^ pair in presence of an acceptor hole due to ${V}_{Zn}$ shows a strong ferromagnetic coupling energy of 50 meV at a relatively large distance of 7.5 Å. Crystal field splitting diminishes the magnetocrystalline anisotropy which results in an anhysteretic magnetization curve at higher doping level. Finally, we show that the overlapping of BMPs causes the alignment of their spins, resulting long-range ferromagnetic order in Cu-doped ZnO. The temperature independence of overlapping region between two consecutive BMPs is responsible for almost temperature independence of magnetization in Cu-doped ZnO and other DMO’s.

## Methods

Cu-doped ZnO films were deposited on the transparent quartz substrate by radio frequency magnetron sputtering. Sputtering targets were prepared by standard solid state route from ZnO and CuO powders (99.999%, Aldrich) weighed in stoichiometry proportions^34,64^. The pressure during deposition was maintained at 1.5 × 10^−2^ *mbar* under Ar/O_2_ (60:40) atmosphere and background pressure was maintained at 1.0 × 10^−7^ mbar. Film thicknesses were in the range of ~1 µm measured by Sopra GES5E spectroscopic Ellipsometer. Magnetic measurements were performed using a Quantum Design Ever Cool MPMS XL-7 superconducting quantum interference device (SQUID) magnetometer with the film orientation parallel to applied field. Grazing incidence x-ray diffraction (GIXRD) studies were performed using PANalytical Xpert Pro system with Cu K_α_ radiation. Optical absorption of the thin films in the range 1000–190 nm was determined using Shimadzu UV-2401PC spectrophotometer. The photoluminescence measurements were performed using an argon ion laser operating at a wavelength of 350 nm with an excitation power of 70 mW. The x-ray photoelectron spectroscopy data were recorded using an Al K_α_ laboratory x-ray source that was operated at 150 W at a chamber base pressure of 5 × 10^−10^ mbar. EPR measurements were carried out at room temperature with Bruker Model EMX MicroX system.

We have performed first-principles DFT simulations using the full-potential Vienna *ab*-initio simulation package (VASP). Projector augmented wave (PAW) potentials were used^65^. Wave functions were expanded in the plane wave basis set with kinetic energy cutoff of 500 eV. For the exchange–correlation functional, we have used GGA with Perdew and Wang (PW91) parameterization^66^. The pure ZnO has the hexagonal closed packed wurtzite structure with theoretically (DFT with GGA) calculated lattice parameters a = 3.28 Å, c = 5.33 Å with u = 0.344. To mimic the 2% doping concentration, we made a 3 × 6 × 3 super cell of wurtzite-ZnO that contains 108 Zn atoms, out of which we randomly chose two Zn atoms and replaced with Cu atoms. The vacancy (~1%) was created by removing one Zn or O atom in between two Cu sites. The large unit cell geometries were further optimized. In our simulations, the forces on each of the atoms were calculated using the Hellmann-Feynman theorem and were subsequently used to perform a conjugate gradient structural relaxation. The structural optimizations were continued until the forces on the atoms converged to less than 0.01 eV/Å.

## Supplementary information

Origin of ferromagnetism in Cu-doped ZnO

## Data Availability

The data that supports the findings of this study are available from the corresponding author on reasonable request.
