# Supplementary material for: Origin of ferromagnetism in Cu-doped ZnO
Source: Sci Rep. 2019 Feb 21;9:2461. doi: 10.1038/s41598-019-39660-x (PMC6385290; doi:10.1038/s41598-019-39660-x)
Supplement: Supplementary file 1 — Origin of ferromagnetism in Cu-doped ZnO [file 41598_2019_39660_MOESM1_ESM.doc]

**Origin of ferromagnetism in Cu-doped ZnO**

Nasir Ali^1^, Budhi Singh^2^, Zaheer Ahmed Khan^3^, Vijya A. R.^4^, Kartick Tarafder^4^ and Subhasis Ghosh^1^

*School of Physical Sciences, Jawaharlal Nehru University, New Delhi, INDIA-110067*

*Inter-University Accelerator Centre, Aruna Asaf Ali Marg-New Delhi, INDIA-110067*

*Semi-Conductor Laboratory, Department of Space, S.A.S. Nagar, Punjab, INDIA*

*Department of Physics, National Institute of Technology Karnataka, Surathkal, INDIA-575025*

**Contents**

**Note 1. Magnetic properties of Cu-doped ZnO films**

**Note 2. Structural investigations of Cu-doped ZnO films**

**Note 3. Optical properties of Cu-doped ZnO films**

**Note 4. Resistivity and dielectric measurements**

**Note 5. Table I**

**Note 1. Magnetic properties of Cu-doped ZnO**

Figure S1 shows the magnetization data of Cu-doped ZnO film with different doping concentration measured at 10 K.

**Figure S1:** Magnetization (M-H) curve of Cu-doped ZnO films with different doping concentrations measured at 10 K.

**Note 2. Structural investigations of Cu-doped ZnO films**

Figure S2 shows the x-ray diffraction (XRD) spectra of undoped and Cu-doped ZnO films. All films show (002) peak at 34.4^o^ corresponding to hexagonal wurtzite ZnO structure. The absence of any peak corresponding to CuO planes indicates homogenous mixing of dopant atom with the host.^1^ Figure 1(b) shows the shift in (002) peak position with different Cu contents in ZnO.

Figure 1(c) shows the lattice constant and stress with different Cu contents in ZnO.

**(a)**

**(b)**

**(c)**

**Figure S2:** (a) X-ray diffraction of undoped and Cu-doped ZnO films. (b). Shifting in (002) peak position with different Cu contents in ZnO. (C) Variation in lattice constant and stress with different Cu contents in ZnO. Connecting lines are guiding for eyes.

**Note 3. Optical properties of Cu-doped ZnO films**

Figure S3 (a) revealed the photoluminescence (PL) spectra of the undoped and Cu-doped ZnO films. The expected emission peak due to the band to band transition in undoped ZnO around ~ 3.4 eV is very weak. The luminescence peak due to zinc vacancy (V_Zn_) at ~ 3.0 eV is very strong indicating that V_Zn_ is prominent in ZnO.^2^ Interestingly, the peak at 3.0 eV disappeared and the luminescence band peaking at ~2.4 eV with zero phonon line at 2.865 eV appeared with Cu doping. This may be due to the formation of Cu_Zn_-V_Zn_ complexes. Huang^3^ et al. also reported that V_Zn_ are crucial for green luminescence in Cu-doped ZnO film. The green emission band has pronounced equidistant peaks spaced in energy by ~ 68 meV corresponding to the longitudinal

optical phonon energy of ZnO. However, the formation of these phonon replicas and the intensity of the green luminescence decrease with Cu doping. Similarly, in the absorption spectra, we have also observed a step around ~ 3.0 eV in the undoped ZnO which gets disappear with Cu doping (Figure S3 (b)).

**(a)**

**(b)**

**Figure S3:** (a) Photoluminescence and (b) absorption spectroscopy of Cu-doped ZnO films.

**Note 4. Resistivity and dielectric measurements**

Figure S4 (a) shows the resistivity *vs* temperature of different Cu-doped ZnO films from 300 K to 20 K. All films show insulator like behaviors with increasing resistivity with decreasing temperature. According to Mott theory, the transport of charges takes place from one localized state to next (energetically favorable) state *via* variable-range-hopping (VRH) Mott’s conduction and is given by^4-6^


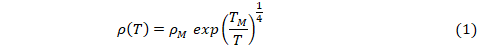


where
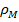
 is a temperature independent resistivity parameter and
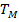
 is a characteristic temperature. By fitting the experimental data with Eq.1 (solid red line) in figure S4(b), we observed that the conduction of Cu-doped ZnO thin films over the temperature range from 180 K to 20 K is governed by Mott’s VRH mechanism which indicates that charge carriers are highly localized to defects sites. The dielectric constant as a function of frequency measured at 300 K is shown in Figure S4 (c).

**(b)**

**(c)**

**(a)**

**Figure S4: (a)** The resistivity *vs.* temperature of Cu-doped ZnO films. All films show insulator behavior. (b) Mott variable range hopping conduction from 180 K to 20 K indicates that carriers are highly localized in Cu-doped ZnO. (c) Dielectric constant *vs*. frequency of ZnO film measured at room temperature.

Figure S5 shows the variation of Cu impurities and separation between them at different Cu contents. Connecting lines are guide for eyes.

**Figure S5:** Variation of Cu impurities and separation between them at different Cu contents. Connecting lines are guide for eyes.

**Table I. XPS peaks position of Cu in Cu-doped ZnO films**

| **Zn_1-x_Cu_x_O** | **Peak position of Cu^2+^**  **(eV)** | **Peak position of Cu^1+^**  **(eV)** | **Area of Cu^2+^** | **Area of Cu^1+^** |
| --- | --- | --- | --- | --- |
| x=0.01 | 953.450 | 951.070 | 484.096 | 214.509 |
| x=0.025 | 954.647 | 952.120 | 429.165 | 1048.759 |

**References:**

1. Z. A. Khan, A. Rai, S. R. Barman, and S. Ghosh, Appl. Phys. Lett. 102, 022105 (2013).
2. H. Zeng, Z. Li, W. Cai, and P. Liu, J. Appl. Phys. 102, 104307 (2007).
3. X. H. Huang, C. Zhang, C. B. Tay, T. Venkatesan, and S. J. Chua, Appl. Phys. Lett. 102, 111106 (2013).
4. Y-L. Huang, S-P. Chiu, Z-X. Zhu, Z-Q. Li, and J-J. Lin, J. Appl. Phys. 107, 063715 (2010).
5. S. Singh, and M. S. Ramachandra Rao, Phys. Rev. B. 80, 045210 (2009).
6. S. Venkatesh, J. B. franklin, M. P. Ryan, J.-S. Lee, H. Ohldag, M. A. Mclachlan, N. M. Alford, and I. S. Roqan, J. Appl. Phys. 117, 013913 (2015).
